# Response to “Toward Audit-Ready Standards for Synthetic Cohorts in Health Decision Science”

**DOI:** 10.34133/hds.0415

**Published:** 2026-01-23

**Authors:** Gregorio Ferreira, Jacopo Amidei, Ruben Nieto, Andreas Kaltenbrunner

**Affiliations:** ^1^ UOC-TECH, Universitat Oberta de Catalunya, Barcelona, Spain.; ^2^ Department of Engineering, Universitat Pompeu Fabra, Barcelona, Spain.

We thank Vijayasimha [1] for the thoughtful and constructive letter regarding our article [2] on large language model (LLM)-generated synthetic population samples and their alignment with real Eysenck Personality Questionnaire Revised-Abbreviated (EPQR-A) psychometric data. We appreciate how the letter situates our findings within the broader and timely discussion on audit-ready standards for synthetic cohorts in health decision science.

Our study aimed to provide an empirical assessment of how closely simulated cohorts produced by a contemporary LLM reproduce real population patterns across multiple languages and demographic strata. In doing so, we found persistent distributional mismatches (e.g., Extraversion/Lie deviations), cross-linguistic variability, and scale-specific reliability limitations (notably for Psychoticism and Lie). These results underscore that synthetic cohorts are not yet surrogate substitutes for real samples in decision-relevant settings and motivate precisely the kind of validation logic and reporting discipline advocated in the letter.

We particularly agree with the 4 needs highlighted by the author. First, synthetic cohorts require structured validation beyond what is typical for predictive models. Our comparison with real EPQR-A test results and our reporting of internal consistency across languages are intended as steps in that direction. Second, provenance and reproducibility are central: we therefore documented model versions, temperature choices, generation pipelines, and full prompting strategies in the Appendix to enable replication and auditability. Third, fitness for purpose should be evaluated in the context of intended use. Our discussion emphasizes that current discrepancies may bias downstream inferences if synthetic cohorts are used prematurely. Fourth, the risks related to the usage of synthetic data indeed warrant explicit stress testing.

We also see the proposed Audit-Ready Synthetic Cohort Standards as a promising organizing framework. From our perspective, a practical next step is to operationalize such standards with a small set of community-accepted baseline checks (e.g., distributional fidelity metrics, subgroup fairness diagnostics, and uncertainty characterization) and to align reporting requirements across journals so that synthetic-cohort studies become comparable and reviewable. We would welcome further interdisciplinary work to refine these checks for LLM-based cohort generation, especially under multilingual conditions where we observed nontrivial variation.

In conclusion, we would like to acknowledge ongoing efforts toward audit-ready standards and believe that our work provides empirical evidence for the need for such standards.
